# Physiological reactions to capture in hibernating brown bears

**DOI:** 10.1093/conphys/cow061

**Published:** 2016-12-15

**Authors:** Alina L. Evans, Navinder J. Singh, Boris Fuchs, Stéphane Blanc, Andrea Friebe, Timothy G. Laske, Ole Frobert, Jon E. Swenson, Jon M. Arnemo

**Affiliations:** 1 Department of Forestry and Wildlife Management, Faculty of Applied Ecology and Agricultural Sciences, Hedmark University of Applied Sciences, Campus Evenstad, NO-2418 Elverum, Norway; 2 Department of Wildlife, Fish and Environmental Studies, Faculty of Forest Sciences, Swedish University of Agricultural Sciences, SE-90183 Umeå, Sweden; 3 Institut Pluridisciplinaire Hubert Curien, Université de Strasbourg, F-67087 Strasbourg, France; 4 CNRS UMR 7178, F-67087 Strasbourg, France; 5 Department of Ecology and Natural Resources Management, Norwegian University of Life Sciences, NO-1432 Ås, Norway; 6 Medtronic Inc., Mounds View, MN 55112, USA; 7 University of Minnesota, Minneapolis, MN 55455, USA; 8 Örebro University, Faculty of Health, Department of Cardiology, SE 70182, Örebro, Sweden; 9 Norwegian Institute for Nature Research, NO-7485, Trondheim, Norway

**Keywords:** Chemical immobilization, ecophysiology, hibernation, research ethics, *Ursus arctos*

## Abstract

Human disturbance can affect animal life history and even population dynamics. However, the consequences of these disturbances are difficult to measure. This is especially true for hibernating animals, which are highly vulnerable to disturbance, because hibernation is a process of major physiological changes, involving conservation of energy during a resource-depleted time of year. During the winters of 2011–15, we captured 15 subadult brown bears (*Ursus arctos*) and recorded their body temperatures (*n* = 11) and heart rates (*n* = 10) before, during and after capture using biologgers. We estimated the time for body temperature and heart rate to normalize after the capture event. We then evaluated the effect of the captures on the pattern and depth of hibernation and the day of den emergence by comparing the body temperature of captured bears with that of undisturbed subadult bears (*n* = 11). Both body temperature and heart rate increased during capture and returned to hibernation levels after 15–20 days. We showed that bears required 2–3 weeks to return to hibernation levels after winter captures, suggesting high metabolic costs during this period. There were also indications that the winter captures resulted in delayed den emergence.

## Abstract (precis)

Researchers around the world capture bears of many species in their winter dens for a variety of purposes. Here, we found that brown bears had an arousal as a result of the captures and their hibernation was disrupted for several weeks.

## Introduction

Wild animals, including brown bears (*Ursus arctos*), are captured for a variety of research and management purposes. Assessing the potential negative effects of these captures is an ethical imperative. Capture of brown bears during the active season is known to have detrimental effects on body condition and to change movement patterns for up to 3–6 weeks after capture (Cattet *et al*., 2008). Although winter capture of American black bears (*Ursus americanus*) is reported to result in fewer injuries than foot snaring (Powell, 2005), capture during hibernation may affect animals differently than capture during the active period, as disturbance during this crucial period can have negative effects on behaviour, habitat use, body condition, foraging opportunities and juvenile survival (Swenson *et al*., 1997). Even the first studies on hibernators reported that disturbance can arouse the animal and ‘frustrate the experiment’ (Hall, 1832). In small mammals, such as bats, arousal during hibernation is energetically costly and thought to decrease winter survival, with body mass lost during hibernation correlating with body temperature and number of arousals (Speakman *et al*., 1991; Boyles and Brack, 2009).

Hibernating ursids undergo an array of physiological changes. In contrast to rodents, bears exhibit a less dramatic drop in body temperature (*T*_b_), protein conservation, absence of urination and defaecation (Hellgren, 1998). During hibernation, both captive and wild brown bears reduce their *T*_b_ by about 3–5°C from active levels of 37.0–37.5°C (Hissa, 1997; Evans *et al*., 2016) and heart rate (H) from about 70–80 beats per minute (bpm) to hibernating levels of around 10–29 bpm (Nelson and Robbins, 2010; Evans *et al*., 2016). These reductions in body temperature and heart rate are connected to the hibernators’ energy savings and reductions in metabolic rate (Geiser, 1988).

In hibernating captive bears, shining a light resulted in raising of the head ~50% of the time [one American black bear, one brown bear and two polar bears (*Ursus maritimus*); Folk *et al*., 1976]. In that study, feeding and watering one of the hibernating brown bears resulted in the recording of HRs at the ‘normal’ level, declining back to hibernation levels after a ‘few’ weeks. Another paper shows a short disruption (1–2 days) to the bear's body temperature caused by blood sampling, but this was not discussed further (Hissa, 1997).

The European brown bear is known to be sensitive to disturbance (Swenson *et al*., 1997). These bears usually den at least 1–2 km from human activity and are tolerant of human activity at this distance. However, activity closer than 1 km and especially within 200 m caused some bears to abandon their dens, especially in the early denning period (Swenson *et al*., 1997). Although abandoned dens were documented to be more frequently located closer to plowed roads (Elfström and Swenson, 2009), the physical characteristics of the denning site did not differ dramatically between successful and abandoned dens (Elfström *et al*., 2008). In Sweden, den abandonment rates are high, with 9% (of 194 bear winters followed by VHF telemetry; Swenson *et al*., 1997) and 22% (of 90 followed by the more accurate GPS technology; Sahlén *et al*., 2015) changing their dens. Although the later study found no gender differences, pregnant adult females that changed dens had significantly greater cub mortality than those that did not abandon dens (60 vs. 6% lost at least one cub at the den or shortly after leaving it). That study further investigated 18 cases of den abandonment and found evidence of human activity in 12 cases and could not exclude human activity in the remainder (Swenson *et al*., 1997). Although both the Scandinavian brown bear and American black bears (Smith, 1986) are reported to find a new den within several weeks of disturbance, in some cases with American black bears, animals remained active after abandonment (Goodrich and Berger, 1994).

Approaching dens on foot has been documented to cause den abandonment (Manville, 1983; Graber, 1990; Goodrich and Berger, 1994; Linnell *et al*., 2000). Smith (1986) found different thresholds for different den types, with tree-denning bears being more tolerant of approach than ground-nesting bears. Den abandonment has also been documented to occur following disturbance by heavy rain and even a pack of hunting dogs (Hamilton and Marchington, 1980) or snowmobile traffic (Elowe and Dodge, 1989). One study with three brown bears exposed to a total of five seismic exploration events reported that in three of five cases the bears responded with increased HR or movement to seismic shots, drilling or vehicle driving at a distance of 1–2 km (Reynolds *et al*., 1983). Another study found increased activity levels when the bears were tracked with VHF receivers from an aeroplane (Schoen *et al*., 1987).

Some authors have reported that both brown and black bears are more susceptible to den abandonment following disturbance earlier in the denning period than later (Tietje and Ruff, 1980; Beecham *et al*., 1983; Smith, 1986; Kolenosky and Strathearn, 1987; Swenson *et al*., 1997; Sahlén *et al*., 2015). Most disturbed bears redenned (Kolenosky and Strathearn, 1987; Hellgren and Vaughan, 1989) and even successfully had cubs (Smith, 1986), although in some cases the cubs died following den abandonment (Elowe and Dodge, 1989; Goodrich and Berger, 1994). Smith (1986) hypothesized that American black bears denning in better-concealed dens were less likely to abandon after capture. Den abandonment is energetically costly, with black bears that changed dens during winter having greater weight loss than undisturbed bears (25 vs. 16% weight loss, respectively; Tietje and Ruff, 1980).

We previously reported that capture of hibernating brown bears resulted in den abandonment in 12 (92%) of 13 captures, compared with 22% overall den abandonment rate in the study area (Sahlén *et al*., 2015). Although we do not know of other reports of the effects of capture on denning brown bears, a study reporting the capture of 14 hibernating female American black bears with cubs found that none abandoned their dens (Doan-Crider and Hellgren, 1996); others have reported that capture or approach of black bears during denning resulted in den abandonment rates of 17% (Tietje and Ruff, 1980) and 29% (Goodrich and Berger, 1994). Based on these studies, the Scandinavian brown bear may be more sensitive to winter disturbance than the American black bear. Here, we used biologgers to document how winter captures affected hibernation patterns, depth and phenology.

## Materials and methods

We captured 15 biologger-outfitted solitary subadult brown bears (2–4 years old; 28–72 kg) in Dalarna County, Sweden between 24 February and 3 March in the years 2011–15. Bears with an expected weight <65 kg were considered manageable and selected for capture (Evans *et al*., 2012). Data on *T*_b_ of undisturbed bears of the same age in the study area were used as a control group (*n* = 11; 43–100 kg). Bears in both groups had been captured by aerial darting in April–May the previous year (Arnemo *et al*., 2012). Bears had previously been fitted with GPS collars (Vectronics Aerospace GmbH, Berlin, Germany) and VHF abdominal implants (Telonics Inc., Mesa, AZ, USA). Eleven captured bears had abdominal *T*_b_ loggers (DST Centi; Star-Oddi, Gardabaer, Iceland) reading at 3 or 4 min intervals; six of these and four additional bears had heart monitors (Reveal^®^XT; Medtronic Inc., Mounds View, MN, USA), which recorded day and nighttime mean HRs; see also Laske *et al*. (2011).

Bears were located for capture in their winter dens using previously deployed GPS and VHF radio collars/implants. Dens were located between 0.3 and 20 km from plowed roads, so when necessary, snowmobiles were used to transport the field team to the den area. We used skis or snowshoes for the last 200–800 m. Once the den was located, a metal grate was placed over the entrance and the bear was darted in the den through the grate using a flashlight and a CO_2_-powered rifle (Dan-Inject^®^, Børkop, Denmark) fired from 0.3–3.5 m. Darts were 3 ml, with a 2.0 mm × 30 mm barbed needle (Dan-Inject^®^). The bears were anaesthetized with medetomidine (Domitor^®^, 1 mg/ml and Zalopine^®^, 10 mg/ml; Orion Pharma Animal Health, Turku, Finland), tiletamine–zolazepam (Zoletil^®^, 500 mg per vial; Virbac, Carros, France) and ketamine (Narketan 10^®^, 100 mg/ml; Chassot, Dublin, Ireland; Table 1). Bears not asleep after 15 min were given a second dart with the same or a half-dose, depending on their initial reaction to the drugs. Once immobilized, we took each of the bears out of the den and placed them on an insulated blanket for monitoring and sampling. Fat, muscle and blood samples were collected and echocardiography was performed for other studies. Afterwards, bears were placed into the dens and the effects of medetomidine were antagnoized with atipamezole (Antisedan^®^, 5 mg/ml; Orion Pharma Animal Health) intramuscularly at 5 mg per mg of medetomidine. The dens were covered with branches and snow and the bears left to recover undisturbed. These methods have been described previously in more detail (Evans *et al*., 2012).

**Table 1:** Body mass, age (in years) and drug doses (in milligrams) used for anaesthesia of brown bears during winter

Sex (age)	Mass (kg)	Year	Variable	Darts	TZ	M	K	Induction
Female (4)	59	2011	*T*_b_	1	63	1.3	75	7
Female (3)	56	2012	*T*_b_	1	63	1.3	75	5
Female (2)	32	2012	*T*_b_	1	32	0.6	37.5	5
Female (2)	30	2012	*T*_b_	1	32	0.6	37.5	11
Female (3)	55	2013	*T*_b_ and HR	1	63	1.3	75	13
Female (3)	52	2013	*T*_b_ and HR	2	94	1.9	112.5	31
Male (2)	40	2013	*T*_b_ and HR	1	31	0.7	37.5	9
Male (2)^a^	54	2013	*T*_b_ and HR	1	63	1.3	75	10
Female (3)	53	2013	*T*_b_ and HR	1	63	1.3	75	8
Female (2)	36	2014	HR	1	63	0.7	37.5	6
Female (2)^b^	32	2014	HR	1	63	0.7	37.5	4
Female (2)^c^	28	2014	HR	1	63	0.7	37.5	4
Male (2)	33	2014	HR	1	63	0.7	37.5	6
Female (3)	45	2015	*T*_b_ and HR	2	188	1.9	112.5	32
Female (3)^a^	72	2015	*T*_b_	1	125	1.3	75	19
mg/kg					1.6 ± 0.8	0.02 ± 0.01	1.4 ± 0.4	
Mean	45 ± 13			1.1 ± 0.4	71 ± 40	1.1 ± 0.4	63 ± 27	11 ± 9

Abbreviations: K, ketamine; M, medetomidine; and TZ, tiletamine–zolazepam. Induction is the time (in minutes) from darting to immobilization.

^a^Denotes the bears that did not change dens after capture.

^b^Denotes one bear that required manual ventilation after respiratory arrest during anaesthesia.

^c^Denotes one bear that was killed and eaten by another bear after den emergence in spring.

To describe the effect of capture on the hibernation pattern, the time between capture and return to hibernation (i.e. the disturbance period) was determined using changepoint analysis (Killick *et al*., 2014) for daily means of both *T*_b_ and daytime HR (08.00–20.00 h). Changepoint analysis detects multiple change points in a time series using a pruned exact linear time (PELT) algorithm, which has increased accuracy over binary segmentation and uses a dynamic programing technique to identify an optimized cost function and the maximal number of segments a time series can be split into (Killick *et al*., 2012). We used the ‘cpt.meanvar’ function from the package ‘changepoint’ in R 3.2.0 (R Development Core Team, 2014). We used the ‘Normal’ as the test statistic and set the penalty value as zero. Summary statistics were calculated during the disturbance period, the week before capture (pre-disturbance period) and the week after the calculated disturbance period (post-disturbance period).

To assess the effect of capture on the depth of hibernation, we used an energy-saving index. We calculated this index as the area under the curve (AUC) from the measured daily mean *T*_b_ curve for three consecutive periods during hibernation; the smaller the AUC energy-saving index, the deeper the hibernation. The AUC was calculated for 21 bears (11 captured, mean body mass 49.7 kg, SE = 3.7 kg; and 10 undisturbed, mean body mass 62.5 kg, SE = 5.4 kg; Table 2). Owing to the maximal body mass of 65 kg that can be handled safely in this capture situation (Evans *et al*., 2012), one bear was in the capture group and 1 year later, when larger, in the control group. We used a Welch two-sample *t*-test to compare the body mass of the captured (early March) vs. the undisturbed group (late April).

**Table 2:** Bears included in the area under the curve (AUC) analysis for body temperature

Identity	Sex	Year	Den captured	Body mass (kg)	Duration (days)	aucdis	aucpre	aucpost
W0818	F	2011	No	48	178	542	530	551
W0820	F	2011	No	57	172	535	530	541
W0824	M	2011	No	74	138	556	542	571
W0904	F	2012	No	63	153	545	536	526
W0910	M	2011	No	55	177	550	537	562
W0910	M	2012	No	100	122	575	556	37
W1017	F	2013	No	64	157	551	535	562
W1205	F	2015	No	75	146	565	546	578
W1316	M	2015	No	43	166	549	533	555
W1317	M	2015	No	46	155	554	534	559
W0825	F	2011	Yes	58	178	582	531	567
W1017	F	2012	Yes	56	167	586	542	577
W1104	F	2012	Yes	30	192	561	521	536
W1104	F	2013	Yes	52	174	588	529	541
W1105	F	2012	Yes	32	199	568	518	536
W1105	F	2013	Yes	55	172	581	533	566
W1110	F	2013	Yes	53	131	580	538	570
W1204	M	2013	Yes	40	176	584	540	560
W1207	M	2013	Yes	54	157	565	535	567
W1304	F	2015	Yes	45	159	578	524	551
W1305	F	2015	Yes	72	158	573	541	562

For the undisturbed bears (*n* = 10; No) body mass was taken during the spring capture following the den exit and for the den-captured bears (*n* = 11; Yes) during the den capture. Duration (in days) from den entry and den exit (start date *T*_b_ < 36.5°C and end date *T*_b_ > 36.7°C). Area under the daily mean body temperature curve for the pre-disturbance (aucpre), the disturbance (aucdis) and the post-disturbance period (aucpost).

To control for the influence of body mass on the AUC, we compared periods of equal length for each period and included all individuals. The length of the mean disturbance period from the changepoint analysis was used as the period length. First, the AUC of the period prior to the disturbance period was analysed to evaluate the validity of the control group. Then the AUC of the disturbance period was compared between the captured and the undisturbed bears. A third period following the disturbance period was analysed to compare the resumption of hibernation in the captured bears. The pre-disturbance period started at day 39 and ended at day 54 (earliest capture) for all bears. The disturbance period included the day of capture and the following 15 days for captured bears. For the non-captured bears, the disturbance period started with the mean day of the year when the captures were carried out (day of year = 58, range 55–61) and the following 15 days. The post-disturbance period started at the end of each bears’ disturbance period. One uncaptured bear with a body mass of 100 kg was excluded from the post-disturbance period; this bear ended hibernation and left the den site within this period.

We compared the AUC for the captured and undisturbed bears during the three periods using the brms package for Bayesian generalized linear mixed models (Buerkner, 2016). The AUC was the response variable; captured or not and the body mass were included as fixed effects and the bear identity (ID) as a random factor. We compared the date of emergence from hibernation for the captured and the undisturbed bears in the same way. For the emergence, the day of the year when *T*_b_ was above 36.7°C (Evans *et al*., 2016) was the response variable. For both models, captured or not and the body mass were fixed factors, and ID and winter (to control for inter-annual variation) were random factors. We set the fixed variables in an interaction and compared with the additive model using the Watanabe–Akaike information criterion (WAIC; Vehtari *et al*., 2015). To support convergence and speed up the modelling process we standardized the body mass by centring the values and dividing them by two standard deviations using the arm package in R (Gelman and Su, 2015). For all models, the priors were set to default and all were run on 4000 effective posterior samples. Plotted model outputs were marginalized over body mass, allowing an interpretation of the average effect of capture given all values for body mass (Buerkner, 2016).

### Ethics

Procedures were approved by the Ethical Committee on Animal Experiments, Uppsala, Sweden (application numbers C47/9, C7/12, C18/15, C212/9 and C268/12), the Swedish Environmental Protection Agency (NV-0758-14) and the Swedish Board of Agriculture (31-11102/12).

## Results

**Figure 1:**
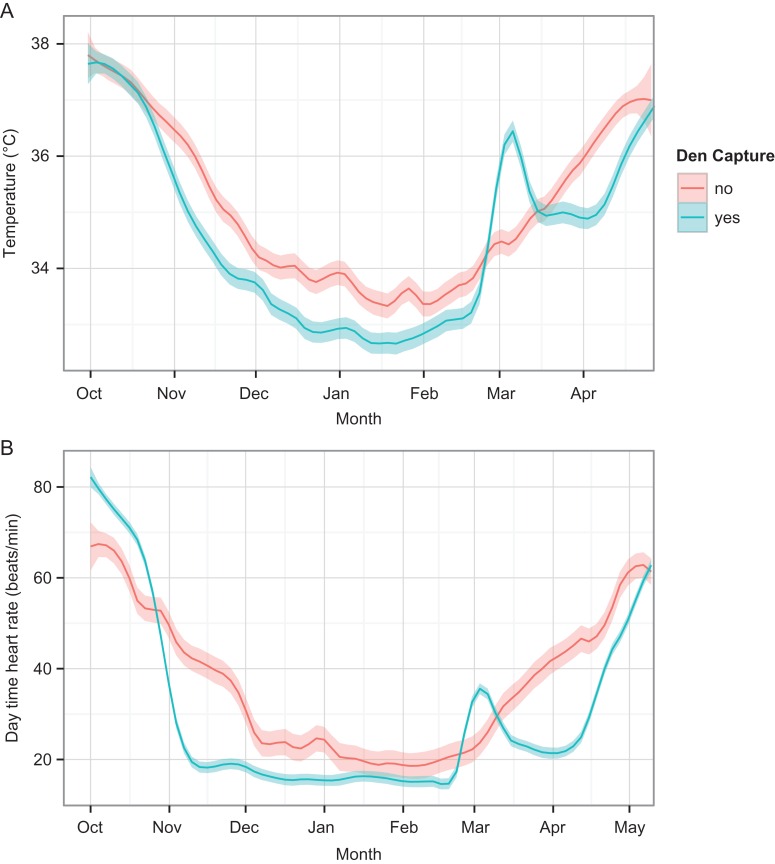
Comparison of physiological variables in captured and undisturbed hibernating brown bears in Sweden. (**A**) Mean daily body temperature of captured (*n* = 11) and undisturbed bears (*n* = 11). (**B**) Mean daytime heart rates of captured (*n* = 7) and undisturbed bears (*n* = 11). The continuous lines show the daily means for individual captured bears with standard errors as shaded areas.

The hibernation pattern between captured and undisturbed bears is visually different for *T*_b_ and HR (Fig. 1). All captured bears aroused to active-level *T*_b_ and HR values. The changepoints between the disturbance and return to normal yielded a mean disturbance period duration for *T*_b_ of 16.1 ± 6.9 days (mean ± SD). For HR, the disturbance period lasted 20.9 ± 6.8 days. Based on GPS positions, only two bears remained at the den site, with 10 and 11 day disturbance periods based on *T*_b_.

**Figure 2:**
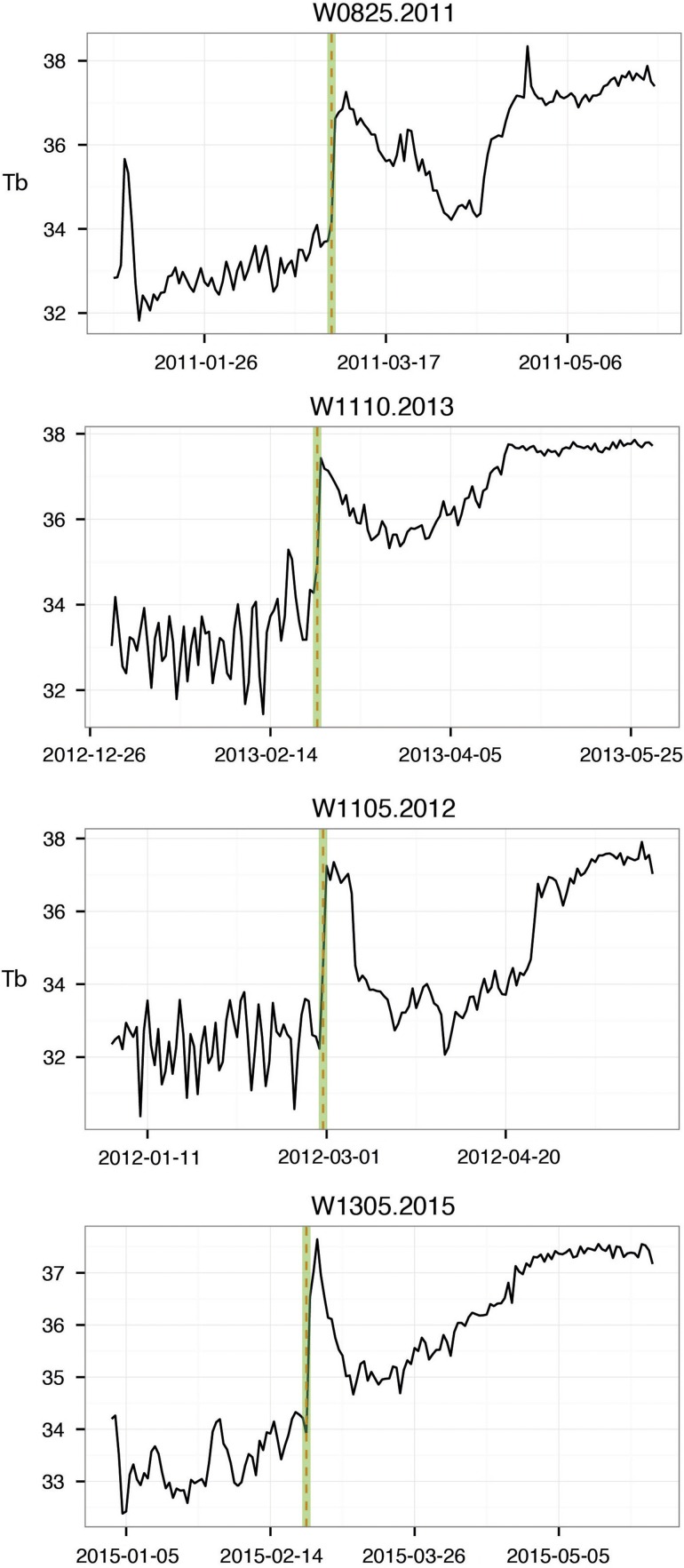
Plots of body temperature of four of the captured bears in this study. The highlight shows the day of capture.

Using the defined periods, captured bears had a *T*_b_ that was 2.6°C higher on average and a HR that was 16 bpm higher during the disturbance period compared with before disturbance. The *T*_b_ and HR did not return to pre-capture levels, but rather to a later phase in the natural physiological rising process shown by the uncaptured bears (examples in Fig. 2). In the post-disturbance period, the captured bears had mean *T*_b_ and HR 1.0°C and 7 bpm higher than in the pre-disturbance period (Table 3). There was no significant difference in body mass between the captured and undisturbed groups, but captured bears tended to be smaller (*t* = 1.9497, d.f. = 16.11, *P*-value = 0.07). The AUC models (included bears are presented in Table 2) that contained an interaction between body mass and treatment did not fit substantially better within any of the periods, according to the WAIC. For the emergence model, the additive model fitted better than the interaction model (WAIC additive, 102.03; SE, 2.82; and WAIC interaction, 115.93; SE, 3.54). In any case, we decided to stick to the simpler additive models for interpretation.

**Table 3:** Descriptive statistics for the brown bears’ daytime mean heart rate (HR; in beats per minute) and daily mean body temperature (*T*_b_; in degrees Celsius) using periods defined by changepoint analysis for each variable

Parameter	Period	Mean	SD	Minimum	Maximum
Daytime heart rate	Period 1	15.35	2.64	10	28
Daytime heart rate	Period 2	31.72	9.32	13	64
Daytime heart rate	Period 3	22.29	4.75	15	47
Body temperature	Period 1	33.6	0.8	30.6	35.3
Body temperature	Period 2	36.0	1.3	32.0	39.3
Body temperature	Period 3	34.6	0.9	32.3	36.8

Period 1 is the week before the capture. Period 2 starts with the day of capture and lasts until heart rate or body temperature reach the hibernation curve again. Period 3 is the week after Period 2. The last four columns show the result of a linear mixed model distinguishing the three periods for each variable.

### Pre-disturbance period

**Figure 3:**
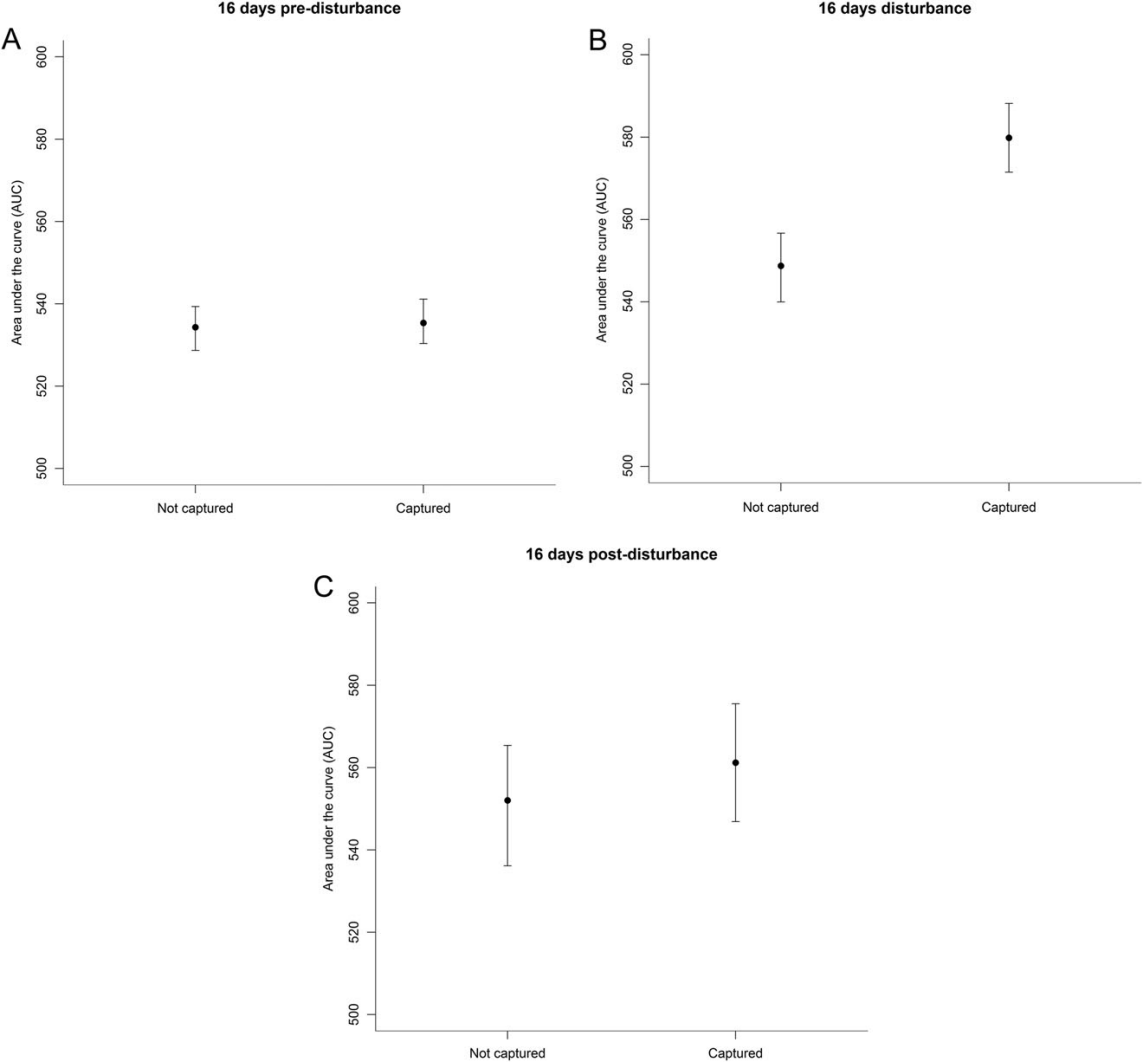
Marginal effect on the energy-saving index (AUC) in the pre-disturbance model testing each brown bear's affiliation to either the control (Not captured) or the test (Captured) group during the pre-disturbance period before the captures (**A**), the disturbance period including the captures (**B**) and the post-disturbance period (**C**). All three model outputs show the average effect of capture given all the values for body mass.

During the pre-disturbance period, body mass had a positive effect on the AUC, meaning that larger bears had an overall higher *T*_b_ than smaller bears. The 95% credible interval of the effect suggested no difference in AUC in the control and the later disturbed group (Fig. 3A and Table 4).

**Table 4:** Model estimates for the pre-disturbance period, the disturbance period and the post-disturbance period, as well as for the day of emergence from hibernation, for hibernating brown bears

Period	Factors	Estimate	SD	Lower CI	Upper CI
Pre-disturbance	Intercept (AUC)	534.27	1.96	530.23	538.03
	Body mass	14.21	2.47	9.41	19.28
	Captured	1.02	2.80	−4.33	6.70
Disturbance	Intercept (AUC)	548.65	3.17	542.27	554.81
	Body mass	14.76	4.55	5.85	23.72
	Captured	31.10	4.57	22.04	40.00
Post-disturbance	Intercept (AUC)	552.23	4.93	542.54	562.17
	Body mass	16.68	6.74	2.89	29.66
	Captured	9.18	6.82	−4.46	22.33
Emergence	Intercept (day of year)	130.38	5.15	118.26	138.95
	Body mass	−0.53	0.07	−0.64	−0.35
	Captured	4.04	2.24	−0.32	9.33

Estimates are the means of the posterior distribution along with the standard deviation (SD) and the 95% credible interval (lower CI and upper CI). Body mass in the disturbance models is centered on zero before fit.

### Disturbance period

During the disturbance period, capture had a positive effect on the AUC, meaning that the overall *T*_b_ curve was raised compared with the undisturbed bears. The effect of body mass remained the same as in the pre-disturbance period (Fig. 3B and Table 4).

### Post-disturbance period

During the post-disturbance period, the effect of capture on the AUC declined. The 95% credible intervals suggested no clear difference between the disturbed and undisturbed bears during this period. The effect of body mass remained similar to that found in both the pre-disturbance and the disturbance periods. The estimate errors and the range of the 95% credible intervals were increased for all values during the post-disturbance period. This reflects the higher between-individual variations during this end phase of hibernation in both groups (Fig. 3C and Table 4).

### Emergence

**Figure 4:**
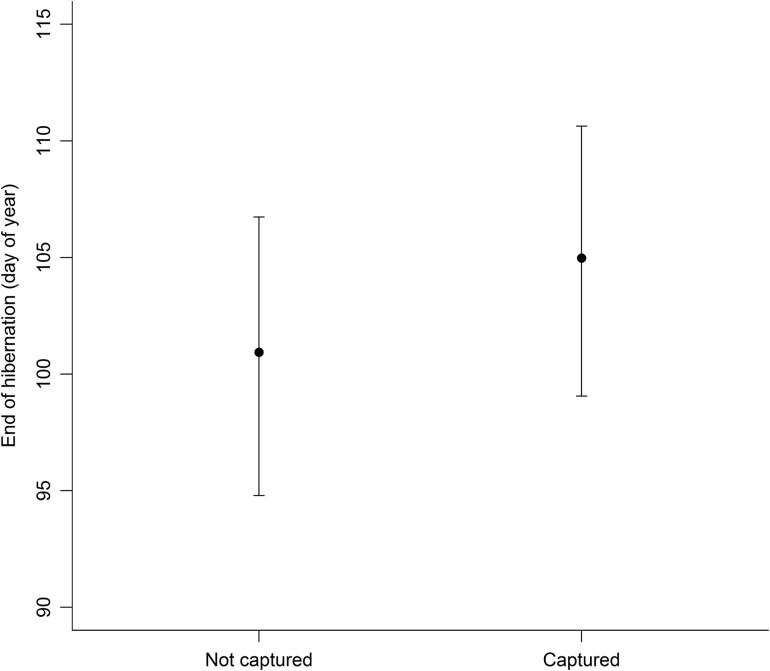
Marginal effect on the day of emergence from hibernation of each brown bear's affiliation to either the control (Not captured) or the test group (Captured). The model output shows the average effect of capture given all values for body mass.

Heavier bears tended to emerge earlier from hibernation. The relatively large 95% credible interval of the effect of capture was close to entirely positive, suggesting a potential delay of emergence from hibernation in the captured group (Fig. 4).

## Discussion

Capture of hibernating brown bears in their dens disrupted their hibernation pattern, causing increased HR and *T*_b_ levels for up to 4 weeks. After this disturbance period, all captured bears returned to hibernation and *T*_b_ patterns did not differ significantly from undisturbed bears. We observed a tendency for delayed emergence (0–9 days) from hibernation of captured bears. However, during this end phase of hibernation, there was also higher inter-individual variation, reflected by the large estimate errors in the post-disturbance model. This suggests that, although hibernation *T*_b_ was disrupted for 16 days, and bears tended to come out a few days later (as seen in studies in black bears, i.e. Hellgren and Vaughan, 1989), the capture events did not substantially affect the hibernation phenology. A similar type of internal set point was observed when an energetic challenge was induced by fasting in thirteen-lined ground squirrels (*Ictidomys tridecemlineatus*) during the annual fattening cycle (Mrosovsky and Fisher, 1970).

In our study, *T*_b_ returned to the hibernation curve faster than the daily mean HR. This is in contrast to a study showing that capture had short-term effects on the HR and activity of American black bears, which usually remain in the same dens after capture (Laske *et al*., 2011). Arousal from hibernation is energetically costly, with the metabolic rate reaching several times the basal metabolic rate (Karpovich *et al*., 2009). One study on edible dormice (*Glis glis*) found that fatter animals aroused more frequently but had a similar length of hibernation, and concluded that surplus energy was used to allow shallower hibernation with more frequent arousal rather than shorter hibernation (Bieber *et al*., 2014). The same study also documented that body mass lost during hibernation correlated with *T*_b_ and number of arousals, concluding that the heavier animals could afford to minimize torpor. This suggests that animals with more fat may tolerate disturbance better and is consistent with reports of shorter hibernation in adult male brown bears (Manchi and Swenson, 2005). In Arctic ground squirrels (*Spermophilus parryii*), arousal episodes are the most energetically costly component of hibernation, accounting for the majority of costs (Karpovich *et al*., 2009). In small hibernators, arousal is defined as a period of euthermia (Karpovich *et al*., 2009). In our study, bears sustained a daily mean *T*_b_ of 36.0°C during the disturbance period, with maximal daily means reaching 39.3°C (Table 3), consistent with this definition. Thus, disturbance probably had high energetic costs during the disturbance period. Unlike the ground squirrels, the bears did not return to their pre-arousal *T*_b_ nor did they have a sustained period of active-level heart rates (Milsom *et al*., 1999).

These results are applicable only to subadult brown bears. Further research would be required to assess the impact of winter captures on other age classes, such as females with cubs or larger bears. However, to the best of our knowledge, larger brown bears have not been captured in the dens because of the assumed high risk to personnel. More detailed studies on metabolic cost are required to draw definite conclusions about the ethical implications of capturing brown bears in the den. The two animals remaining at the den site had shorter disturbance periods, so future research could be targeted to investigating whether certain den types might result in less den abandonment. Also, it seems that, based on lower post-capture abandonment rates (0–17%), the American black bear may be less sensitive to winter captures and other types of winter disturbance (Tietje and Ruff, 1980; Goodrich and Berger, 1994; Doan-Crider and Hellgren, 1996). It is not possible say whether these differences are species related or result from historical differences in human pressures.

Much of the variation in duration and depth of hibernation among individuals was attributed to body mass. Such an importance of body mass for the depth of hibernation has also been established for small species (Geiser, 2004). This fact made the comparison difficult, as the captured bears tended to have lower body mass compared with the undisturbed bears. The reason for the difference in body mass is the lack of undisturbed bears within the size range where den capture was possible. We tried to control for the effect of body mass using the marginalizing techniques implemented in the Bayesian regression models. In addition, the assignment to either group was not detectable before the disturbance, and we could therefore compare these two groups. Our findings imply that den captures have energetic costs during arousal and the subsequent period of euthermia.
